# The EANM practical guidelines for sentinel lymph node localisation in oral cavity squamous cell carcinoma

**DOI:** 10.1007/s00259-018-4235-5

**Published:** 2018-12-18

**Authors:** Francesco Giammarile, Clare Schilling, Gopinanth Gnanasegaran, Chandrasckhar Bal, Wim J. G. Oyen, Domenico Rubello, Thomas Schwarz, Girolamo Tartaglione, Rodolfo Nuñez Miller, Diana Paez, Fijis W. B. van Leeuwen, Renato A. Valdés Olmos, Mark McGurk, Roberto C. Delgado Bolton

**Affiliations:** 1 Nuclear Medicine and Diagnostic Imaging Section, Division of Human Health, International Atomic Energy Agency, PO Box 100, 1400 Vienna, Austria; 2 Department of Head and Neck Surgery, University College Hospital, 235 Euston Road, London, NW1 UK; 3 Department of Nuclear Medicine, Royal Free London NHS Foundation Trust, Pond Street, London, NW3 2QG UK; 4 Department of Nuclear Medicine, All India Institute of Medical Sciences, New Delhi, 110029 India; 5 Department of Nuclear Medicine, The Institute of Cancer Research and The Royal Marsden NHS Foundation Trust, London, UK; 6 Department of Nuclear Medicine, Radiology, and Clinical Pathology, Rovigo Hospital, Rovigo, Italy; 7 Division of Radiology, Department of Nuclear Medicine, Medical University Graz, Graz, Austria; 8 Nuclear Medicine, Cristo Re Hospital, Rome, Italy; 9 Interventional Molecular Imaging Laboratory and Nuclear Medicine Section, Department of Radiology, Leiden University Medical Centre, Leiden, The Netherlands; 10 University Hospital San Pedro and Centre for Biomedical Research of La Rioja (CIBIR), Logroño, La Rioja, Spain

**Keywords:** Sentinel node, Imaging, Oncology, Oral cavity, Squamous carcinoma, Radiotracer, Colloid, SPECT, SPECT/CT, Radioguided surgery, Intraoperative gamma camera, 3D imaging, International Atomic Energy Agency

## Abstract

**Purpose:**

Sentinel lymph node biopsy is an essential staging tool in patients with clinically localized oral cavity squamous cell carcinoma. The harvesting of a sentinel lymph node entails a sequence of procedures with participation of specialists in nuclear medicine, radiology, surgery, and pathology. The aim of this document is to provide guidelines for nuclear medicine physicians performing lymphoscintigraphy for sentinel lymph node detection in patients with early N0 oral cavity squamous cell carcinoma.

**Methods:**

These practice guidelines were written and have been approved by the European Association of Nuclear Medicine (EANM) and the International Atomic Energy Agency (IAEA) to promote high-quality lymphoscintigraphy. The final result has been discussed by distinguished experts from the EANM Oncology Committee, and national nuclear medicine societies. The document has been endorsed by the Society of Nuclear Medicine and Molecular Imaging (SNMMI).

These guidelines, together with another two focused on Surgery and Pathology (and published in specialised journals), are part of the synergistic efforts developed in preparation for the “2018 Sentinel Node Biopsy in Head and Neck Consensus Conference”.

**Conclusion:**

The present practice guidelines will help nuclear medicine practitioners play their essential role in providing high-quality lymphatic mapping for the care of early N0 oral cavity squamous cell carcinoma patients.

## Introduction

The accurate harvesting of a sentinel lymph node (SLN) in oral squamous cell cancer entails a sequence of procedures with components from different medical specialties, including nuclear medicine, radiology, surgery, and pathology. The topics covered are presented under the headings:

- Goals
- Background and definitions
- Indications
- Procedure success rate, and qualifications and responsibilities of personnel
- Procedures in nuclear medicine
- Procedures in the surgical suite
- Radiation dosimetry
- Issues requiring further clarification

## Goals

The present practice guidelines have been prepared for nuclear medicine practitioners. The intention is to help optimize the diagnostic information that can be obtained from SLN procedures. If specific recommendations cannot be based on evidence from original scientific studies, referral is made to “expert opinion” and similar expressions. The recommendations are designed to assist in the referral, performance, interpretation, and reporting of the SLN procedure.

## Background and definitions

### Oral squamous cell cancer

Oral squamous cell cancer (OSCC) is one of the most common cancers worldwide, accounting for more than 264,000 new cases and 128,000 deaths annually [1, 2]. Three-quarters of affected people are in the developing world, while in developed countries OSCC is the eighth most prevalent form of cancer. Determining the presence or absence of nodal metastases is of paramount importance for staging, treatment planning, and prognosis. The incidence of occult metastases in patients with clinically node-negative OSCC is high, with many series reporting rates greater than 30% [3–6]. SLN procedures provide a realisable means for mapping the most likely lymphatic tumour dissemination pathways and thus aid the identification of early lymphatic lymphatic spread (micrometastases). Cervical lymph node involvement is the most important prognostic factor for patients with OSCC [6–8].

Elective treatment of the clinically node-negative neck remains a controversial topic. Over the last 2 decades, much work has been undertaken to identify reliable predictors of occult metastases, of which tumour depth appears to be the best available [9–12]. However, the predictive value of tumour depth and other primary tumour characteristics is still insufficient to negate the need for surgical staging of the cervical node basin [13, 14]. Thus, elective neck dissection (END) is the current standard of care [15]. Unfortunately, the invasive nature of END increases the chance of surgery-induced side-effects that impair the patient’s quality of life [16]. Further, END results in an error rate of approximately 10% in the ipsilateral neck [17]. In practice, END provides valuable prognostic information regarding nodal status and simultaneous treatment of those patients found to be pathologically node-positive. Previously, ENDs invariably took the form of a modified radical neck dissection; however, there is increasing evidence that selective neck dissection is as efficacious as comprehensive neck dissection in treating the node-negative neck [3, 18–24]. The shift towards more conservative surgical procedures has occurred primarily in the last 2 decades; this shift was facilitated by the work undertaken by Lindberg [25], Byers et al. [26], and Shah et al. [4]. They described that the lymphatic dissemination of rogue tumour cells often is related to the same anatomical patterns of lymphatic drainage. Knowledge of these patterns has made it possible by way of an educated gamble for the extent of neck dissections to be progressively limited to those nodal levels at highest risk. Sentinel lymph node biopsy (SLNB) represents an extension of this philosophy of limiting neck surgery but is quite different, as it objectively identifies the presence of neck metastasis and bypasses the educated gamble. Thus, SLNB can be regarded as a surgical staging test.

The aim of this guideline is to provide evidence-based guidelines for the use of SLNB as a staging tool in patients with early OSCC, presenting the best available evidence at the time of writing. The existing literature was reviewed, utilizing electronic techniques (Medline, Best Evidence, the Cochrane Library, Dare) and manual searching techniques. Where little or no data existed from randomized controlled prospective trials, emphasis was given to data from large series or reports from recognized experts in the field. It is recognized that higher-level evidence from future studies may modify the recommendations made in these guidelines.

### Definition of a sentinel lymph node

The SLN concept states that the spread of a malignant tumour is stepwise and embolic in nature, via the lymphatic vessels to the first-echelon lymph node(s) encountered in the corresponding regional draining basin. These lymph nodes most probably harbour occult metastases, and are designated the SLN. Excisional biopsy and pathological evaluation of the SLNs therefore allows prediction of the disease status of the remaining cervical lymph node basin, avoiding the need for a neck dissection in the case of a negative result. SLNs need not necessarily be those closest to the primary tumour, and there may be multiple SLNs [25]. In theory, ^18^F-FDG PET might be a method of identifying these early deposits but clinically node-negative patients often contain lymphatic micrometastases (< mm), without a structured vascular network [27], which impairs lesion identification. Hence, diagnostic approaches aimed at identifying micrometastases are based on establishing a patient-specific means to map the lymphatic drainage pathways from the tumour. To this end, radiocolloids are locally administered, following by their lymphatic effluence [28]. In this process, the radiocolloids “target” the lymph nodes, where they reside for longer periods of time. Early effluence from the tracer deposition sites can be visualized using early dynamic lymphoscintigraphy (LSG); lymphatic channels are usually visualized, and lymph nodes on a direct drainage pathway may be distinguished. Later images visualize the (S)LNs that accumulated the tracer.

Lymphatic mapping and SLNB were first reported by Cabañas for penile cancer in 1977 [26]. In 1992, Morton et al. [25] were the first to describe the use of intradermal isosulfan blue dye injection for intraoperative SLN localization in patients with malignant melanoma subsequently to preoperative lymphatic mapping using cutaneous tracer injection. The approach of Morton and colleagues, requiring imaging before surgery to determine which lymph node stations were at risk to harvest metastasis from the primary tumour, assumed individual variability in lymphatic drainage, thus differing from the concept of Cabañas, which was based on searching for the SLN in a fixed site (specifically along the superficial epigastric vein in the groin). The year after the publication by Morton et al., Alex et al. [4] described a peritumoral intradermal injection of radioactive tracer (^99m^Tc-sulphur-colloid), integrating preoperative imaging and intraoperative gamma probe radiolocalization of SLNs in just one procedure. The SLN concept has since been extensively studied and validated for patients with cutaneous melanoma [27] and breast cancer [28], and studies to date have indicated a high level of accuracy in patients with OSCC [29, 30].

For SLNB in OSCC, the sensitivity ranges between 64% [29] and 100% [30] in single-centre studies, but the accepted range for multicentre studies and meta-analysis is in the range of 82.5%–88% [31–33].

## Indications

In general, SLNB is indicated for patients with biopsy-proven OSCC staged as early tumours with clinically (palpation) and radiologically (ultrasound, CT, or MRI) N0 neck. The most common sites are the lateral border of the tongue and anterior floor of mouth [33–35]. In addition, early OSCC tumours of the oral cavity such as the gingiva, hard and soft palate, retromolar area, lip, buccal mucosa, or sulcus are candidates for SLNB.

The current indications for SLNB are:

- To stage the ipsilateral, and eventually contralateral, N0 neck in patients with a lateralised primary tumour.
- To assess bilateral N0 necks in primary tumours close to, or crossing, the midline.

The prerequisites for the patients to be eligible for SLNB procedure are described in Table 1 and elaborated on in the surgical guidelines. The precautions and potential limitations are described in the section “Issues requiring clarification“ below.

**Table 1:** Prerequisites for sentinel node biopsy

Prerequisites for sentinel node biopsy	
Prerequisites	Discussion
Patients should also be fit enough preoperatively to withstand a subsequent neck dissection if the SNB is positive	The overall health of the patient should be carefully evaluated prior to SLNB to determine if they have sufficient reserve to undergo two surgical procedures within a short period of time. If there is doubt, it is preferable to undertake END over SLNB.
Pre-operative staging of the neck	Patients should undergo clinical and radiological staging in accordance with national guidelines and results reviewed within local multidisciplinary team (MDT) setting [36].
Only patients with N0 neck should be offered SLNB	Criteria for N0 neck on US, CT, and/or MRI are nodes measuring < 1.1 cm or up to 1.5 cm in level II and no atypical features. Nodes that are considered borderline can be further assessed by ultrasound-guided fine needle aspiration cytology or FDG PET/CT.

## Injection technique

The most common radiotracer in Europe is ^99m^Tc-labelled nanocolloids. Other radiotracers will be discussed in the last section of this paper. The total injected activity varies depending on the protocol, recommending 40–50 MBq for a single-day protocol, whereas 70–120 MBq should be injected for 2-day protocols, resulting in sufficient remaining activity for intraoperative detection via gamma tracing/imaging. The aforementioned activity might be diluted in a total volume not exceeding 0.4–0.5 mL (0.1–0.2 mL each syringe) of saline solution.

To reduce the pain of the injection and also reduce the risk of patient movement during the injection, local anaesthesia is recommended in the form of lidocaine hydrochloride 10% spray or gel, or a high lingual nerve block (injection just below the mucosa behind the 3rd molar tooth, distant from the tumour site).

The radiotracer should be administered with two to four superficial (submucosal) injections, depending on the size and localization of the tumour, in the cardinal points around the cancer at 3, 6, 9, and 12 o’clock. This said optimization of injection procedures, e.g., by performing injections that target the tumour or the deep periphery of the tumour, may potentially yield a better 3D coverage of the lymphatic effluence.

Immediately after injection, the patient should rinse the oral cavity without swallowing in order to reduce oral uptake of non-injected radiotracer and avoid saliva contamination.

## Preoperative imaging

The acquisition protocol should include the following:

1. Positioning: adequate anterior hyper-extension of the neck is required, as long as tolerated by the patient, in order to accurately evaluate the neck levels, especially level I.
2. Dynamic acquisition: acquire immediate dynamic images of the neck in anterior view, (within 5 min post-injection), the dynamic study encompassing at least the first 10–15 min. This part of the imaging process is used to identify lymphatic vessels that drain the tumour.
3. Early static images: anterior and lateral views should be acquired shortly after dynamic acquisition. Both of the above-described acquisition steps are particularly important in order to visualize the lymphatic pathways, which makes it possible to distinguish between SLNs and second-tier lymph nodes. To improve the quality of imaging, the distance between the collimator and the neck should be minimal. Also, an adequate zoom-factor (× 1.5) should be used. This part of the imaging process is used to identify the LNs that receive direct drainage from the tumour.
4. Late images: a late static image at 60–120 min post-injection using the same views as in the early static images should be acquired, at least anterior views if SPECT/CT is also performed. This part of the imaging process is used to identify additional LNs that receive a somewhat slower direct drainage from the tumour. But importantly, comparing early and late images provides a crucial tool that is needed to distinguish between the surgically relevant SLNs and irrelevant higher echelon nodes, which can be considered as false positives of the SLN procedure.
5. Skin marking is recommended to guide the surgeon to search SLNs, as per local protocol.
6. SPECT/CT: when available, SPECT/CT of the neck is mandatory. It should be performed immediately after the late static images. SPECT/CT provides accurate anatomical localization and depth evaluation of SLNs.
7. Count rate: duration of all image sets (dynamic, static, and SPECT) should be long enough to yield a statistically significant number of counts that assures high-quality imaging. As such, lower degrees of draining may be compensated by the use of more sensitive SPECT imaging.
8. Collimation: optimal collimators should be used on the gamma camera to avoid potential star artefacts that obscure SLNs in close proximity to the injection site.

The following tables (Table 2 and Table 3) describe the optimal parameters for image acquisition.

**Table 2:** Optimal imaging parameters for planar dynamic and static image acquisition

Optimal imaging parameters for planar dynamic and static image acquisition	
Issue	Optimal parameter
Acquisition	Image immediately after injection
Positioning	Minimize neck–collimator distance
Energy	140 KeV, 20%
Matrix acquisition	128 × 128
Preset time	120–300″ (s)
Zoom factor	× 1.5
Collimator	LEGP or equivalent, depending on the manufacturer

**Table 3:** Optimal imaging parameters for SPECT/CT acquisition

Optimal imaging parameters for SPECT/CT acquisition	
Issue	Optimal parameter
Acquisition	Image at time point with maximum expected signal to background ratio
Orbit	Circular (minimize patient detector distance)
Angle steps	3°
Views	120 (60 × 2 dual-head camera)
Pixel matrix	128 × 128
Time per view	20–30″ (s) per view
Collimator	LEHR or equivalent, depending on the manufacturer

## Imaging report

**Fig. 1:**
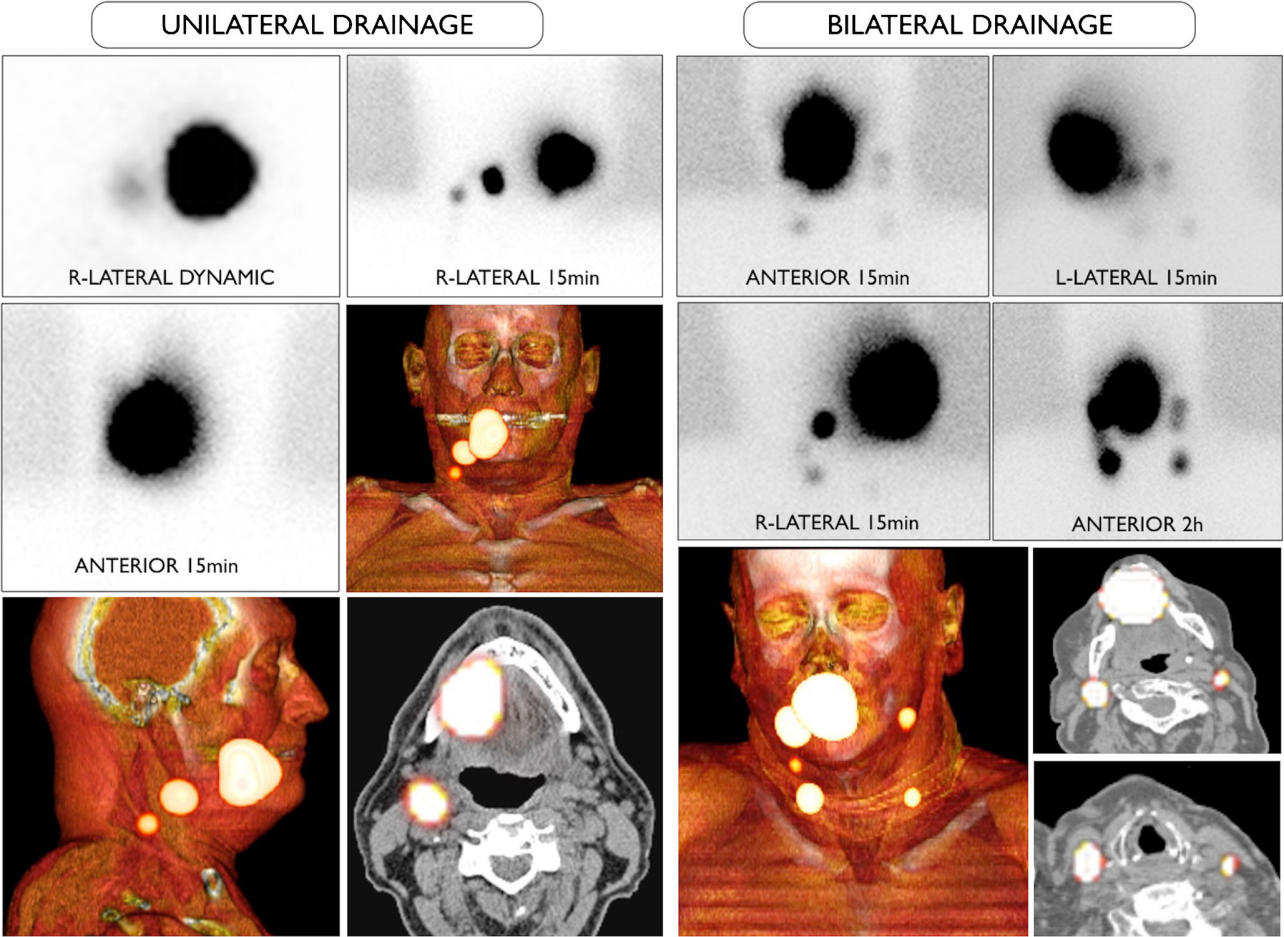
Lymphatic drainage to the neck is unilateral in a 55-year-old male patient with a T1 primary tumour localized on the right side of the tongue (on the *left*) and bilateral in a 72-year old female patient with a T1 midline tongue carcinoma (on the *right*). Note that in both cases the sternocleidomastoid muscle, as depicted on volume rendering and cross-sectional SPECT/CT, is an excellent landmark to anatomically refer the location of sentinel lymph nodes in relation to lymphatic basin and surgical neck level

**Fig. 2:**
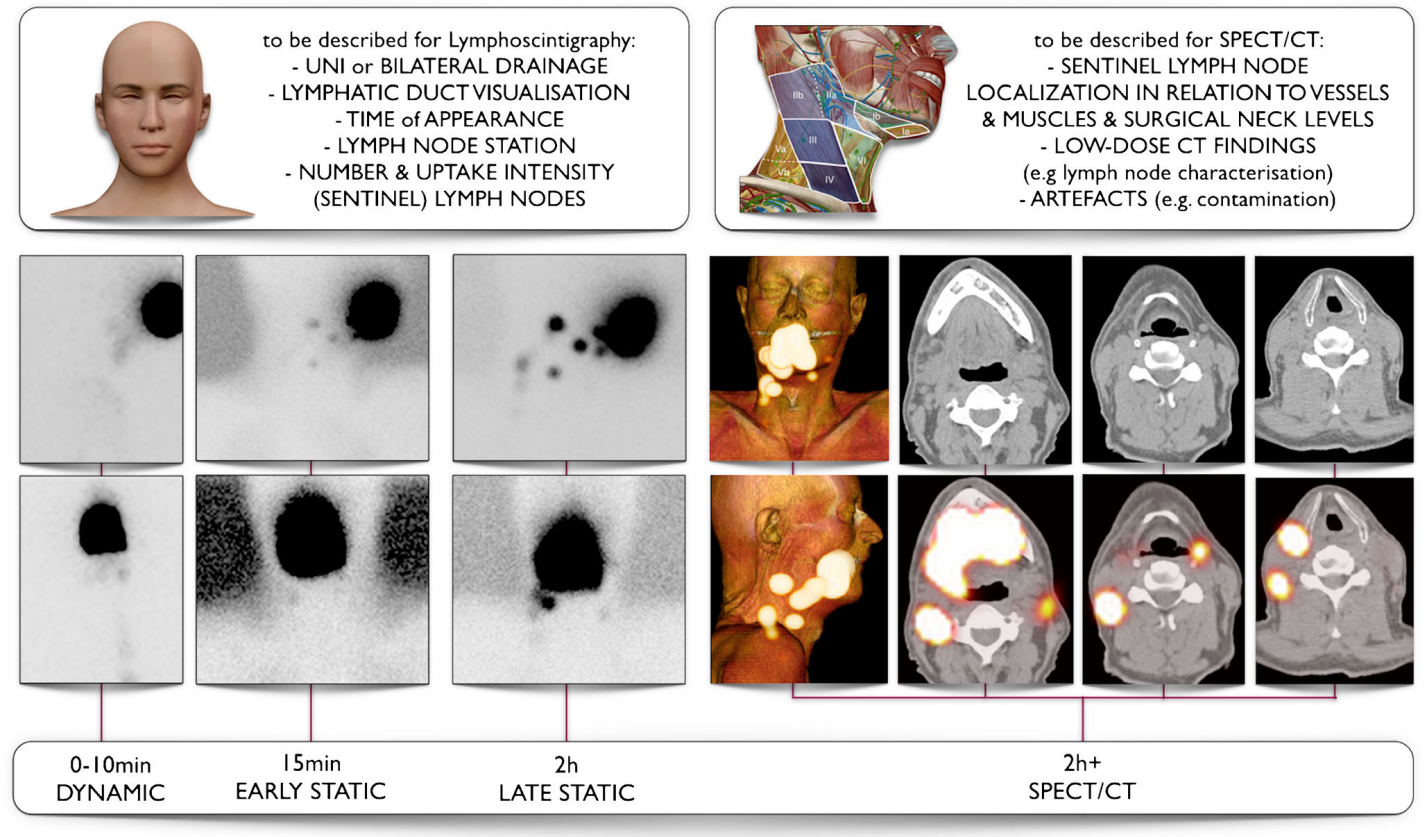
Schematic imaging report generation with a summary of interpretation criteria for lymphoscintigraphy (on the *left*) and SPECT/CT (on the *right*) for a 61-year-old male patient with a T2 midline floor of mouth carcinoma. On dynamic images there is only visualization of activity along throat and oesophagus but no evident lymph node uptake. By contrast, on early static images initial lymph node uptake on the right side of the neck is seen with increasing intensity on delayed static images, which also show drainage to the left side. On SPECT/CT sentinel lymph nodes in level II and III of both sides of the neck are seen whereas the radioactivity just behind the injection site on the right is associated with internal contamination along the oropharynx

A nuclear medicine report has to include several factors such as: (a) visualisation of lymphatic ducts, (b) order of appearance, (c) lymph node basin and (d) intensity of lymph node uptake [37]. On the basis of these factors, visualised radioactive lymph nodes may be considered *definite* SLNs (when lymph nodes draining from the site of the primary tumour are visualised with their own afferent lymphatic vessel or when a single radioactive lymph node in a lymph node basin is seen) [38], *highly probable* SLNs (when lymph nodes appear between the injection site and a first draining node or when nodes with increasing uptake appear in other lymph node stations), or *less probable* SLNs (second-echelon lymph nodes in the head and neck). These aspects are highlighted in Figs. 1 and 2.

In general, early planar imaging helps to identify the order of drainage. First, draining lymph nodes as SLNs by visualisation of lymphatic ducts (Fig. 1). These lymph nodes can be distinguished from second-echelon lymph nodes, which mostly appear on delayed planar images. In other cases, a single lymph node is seen on early and/or delayed images. This lymph node is also considered a definite SLN. Due to its increased sensitivity, SPECT/CT may detect additional lymph nodes in other basins such as the contralateral side of the neck. It should be emphasised, particularly in the contralateral neck even if the signal is relatively weak, that these lymph nodes might be considered highly probable SLNs. Less frequently, a radioactive lymph node may appear later and more proximal to the oral cavity injection site than a first draining node; its increasing uptake on delayed images can confirm this lymph node as a highly probable SLN and help to distinguish it from prolonged activity at a lymphatic duct valve, which usually shows decreasing intensity on delayed images [39]. In specific cases, contrast enhanced (ce) SPECT/CT, or image fusion with ceCT or ceMR, may provide an additional value, at the expense of increased complexity of the procedure. Recently, a new MRI technology called D-Prep MRI was introduced based on its ability to more clearly delineate LNs [40].

For reading/reporting purposes, SPECT/CT images are mostly displayed in a manner similar to that of conventional tomography. The two-dimensional display of fused images to be correlated with CT and SPECT is facilitated by multiplanar 3D reconstruction, and the use of cross-reference lines allows navigation between axial, coronal, and sagittal views. At the same time, this tool enables the correlation of radioactive SLNs seen on fused SPECT/CT with lymph nodes seen on CT. Most frequently, a radioactive SLN corresponds to a single lymph node on CT, but in some cases it correlates with a cluster of lymph nodes. This information may be useful for the intraoperative procedure and the post-excision control using portable gamma cameras or probes, as more radioactive SLNs may be harvested at the same location.

The use of maximum intensity projection (MIP) to display fused SPECT/CT images may also help surgeons to anatomically recognise and localise SLNs. MIP is a specific type of rendering in which the brightest voxels are projected into a three-dimensional image. A limitation of MIP is that the presence of other high-attenuation voxels on CT may make it difficult to recognise the vasculature and other anatomical structures. Further, MIP provides a two-dimensional representation, which cannot accurately depict the actual relationships of the vessels and other structures. SLN localization in a three-dimensional context can also be supported by the application of volume rendering (VR). In this modality, different colours are assigned to anatomical structures such as vessels, muscles, bones, and skin. This results in easily recognisable anatomical reference points facilitating the localisation of SLNs, for instance, in relation to the vasculature. By incorporating a colour display, VR improves visualisation of complex anatomy and 3D relationships, facilitating correlation with cross-sectional analysis. Interpretation of the VR images is more intuitive than that of axial cross sections and helps identify the anatomical context of the SLNs. It is also very helpful to the surgeon if the nuclear medicine report indicates the position of the SN in relation to local anatomical structure. This is normally local muscles such as the bellies of digastric or omohyoid, and in particular the jugular vein. The lymphatic drainage from oral cancer is invariably to nodes along the jugular vein. SNs posterior to the jugular vein and omohyoid muscle can be difficult to find through a limited access incision, and it is particularly helpful to be aware of their presence prior to surgery.

Identification and localization of SLNs in the nuclear medicine report need to be related to the anatomical and surgical approach. According to the American Joint Committee on Cancer (AJCC)–Union for International Cancer Control (UICC) TNM (tumour, node, metastases) staging system, the lymph nodes in the neck may be subdivided into specific anatomic subsites and grouped into seven levels in each side of the neck (Fig. 2). Level I includes the submental (sublevel Ia) and submandibular (sublevel Ib) lymph nodes. Level II contains the upper jugular lymph nodes that extend from the inferior border of the hyoid bone to the base of the skull; in relation to the vertical plane delineated by the spinal accessory nerve, the lymph nodes located anteriorly (medial) constitute sublevel IIa, and the nodes located posteriorly (lateral) correspond to sublevel IIb. Level III includes the middle jugular nodes (cranial to the cricoid), and level IV the lower jugular nodes (caudal to the cricoid). The posterior border of regions II, III, and IV is the posterior border of the sternocleidomastoid muscle, which is the anterior border of level V. This latter group is composed of the sublevels Va (which includes the spinal accessory nodes) and Vb (which includes lymph nodes along the transverse cervical vessels) and the supraclavicular lymph nodes (with the exception of the Virchow node, which is located in level IV). Level VI contains the pretracheal and paratracheal nodes, the precricoid Delphian node, and the perithyroidal nodes, including the lymph nodes along the recurrent laryngeal nerves. Finally, level VII includes the superior mediastinal lymph nodes [41, 42].

Summarizing, each component of the lymphatic mapping procedure needs to be described incorporating the above-mentioned criteria (Fig. 2).

For dynamic studies, define and describe:

- Lymphatic ducts directly draining from the injection site to lymph nodes.
- Bilateral or unilateral drainage.

For early and delayed static images:

- Number and intensity uptake of first-echelon nodes.
- Number of second-echelon nodes.
- Additional lymph nodes appearing on delayed images in other basins.

For SPECT/CT:

- Identification of additional SLNs.
- Localization of SLNs with description of lymph node stations and reference to anatomical landmarks (vessels and muscles). Based on the CT images, the likeliness of a nodal cluster behind a single hot-spot should be indicated.
- Localization in reference to surgical neck levels (I to VII right and left).
- Secondary findings on low-dose non-enhanced CT include enlarged nodes, cluster of nodes, incidental findings, and other abnormalities.

**Fig. 3:**
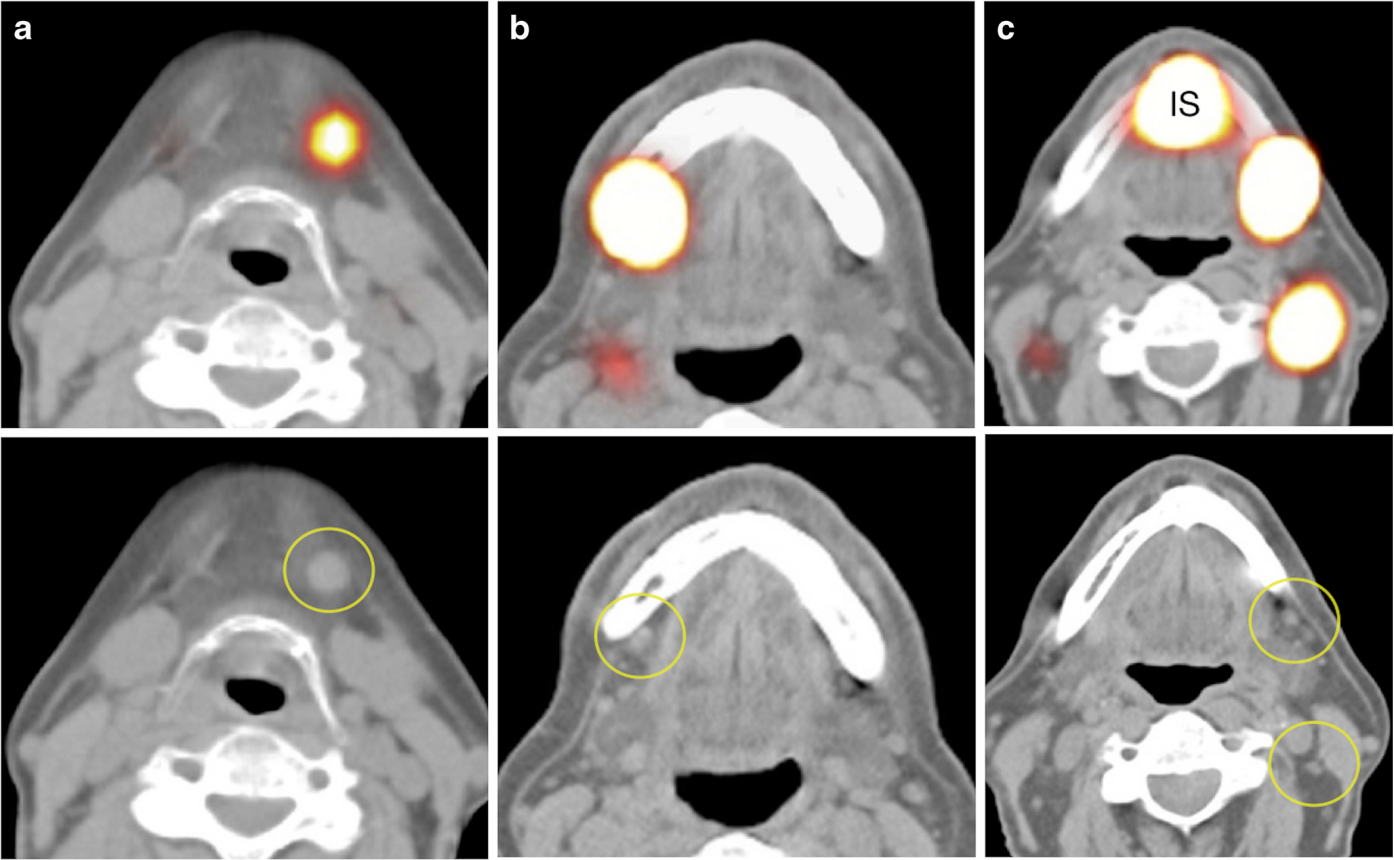
Importance of SPECT/CT and low-dose (ld) CT in characterising sentinel lymph nodes (SLN) in the vicinity of primary tumours in oral cavity. On the *left* (**A**) SPECT/CT (*top*) showing uptake in a SLN in level 1a on the left, which corresponds with an enlarged lymph node (*circle*) on ldCT (*bottom*). On *middle* (**B**) SPECT/CT (*top*) shows intense uptake in a SLN in level 1b on the right, whereas on corresponding ldCT (*bottom*) a slightly elongated lymph node (*circle*) is seen. Finally, on the *right* (**C**) SPECT/CT (*top*) shows drainage from the injection site (IS) to SLNs respectively corresponding with a lymph node cluster in level Ib and a single node in level II of the left side of the neck (*circles*) on ldCT (*bottom*)

The conclusion of the report should include:

- Number, uptake intensity, and anatomical localization of SLNs related to lymph node station and surgical level of each side of the neck.
- Order of appearance of nodes.
- Cutaneous marking: description of the localization of the SLNs.
- SPECT/CT labelling based on anatomical landmarks (Fig. 3).
- Volume-rendered SPECT Images.
- Second-echelon lymph nodes considered as low-probability SLN.

Selected images including dynamic, static, and 3D SPECT/CT images as described, in which the SLNs are indicated, should be uploaded to the PACS (or printed if PACS is not readily available to the multi-disciplinary team).

## Dosimetry and radiation protection

The use of radioactive colloids for SLNB requires the optimization of radiation safety issues, including issues regarding patients, staff in nuclear medicine departments, the operating room, pathology laboratories, and the disposal of radioactive waste. Presently available dosimetric data are derived from the breast cancer and melanoma SLNB literature, where the absorbed doses to patients are determined to be low [43, 44]. While no specific OSCC data exist, for the ^99m^Tc-based radiocolloids, when applied at the same or a similar activity, the radiation risk associated with this procedure is low [45]. This may, however, change when radiocolloids are used that have, for example, ^89^Zr as radiolabel [46, 47].

### Patients

Lymphoscintigraphy is a procedure involving low activities. The estimated local radiation dose varies depending on the administered activity, injection site, volume of tracer, the use of multiple injections, and retention time.

The different radiopharmaceuticals used for SLN imaging show minor differences in dosimetry. The local absorbed dose at the injection site with respect to the most common radiocolloids is less than 50 mGy/MBq [48, 49]. In determining the effective dose, it should be considered that the radiolabelled colloid migrates minimally throughout the bloodstream or reticuloendothelial system (RES) or beyond the SLN and second-echelon lymph nodes. Assuming that 20% of the administered activity is absorbed in the RES systemically, the effective dose is calculated as 2 μSv/MBq in a ‘worst-case’ calculation for melanoma [50]. This corresponds to 0.04 mSv after an injection of 20 MBq of ^99m^Tc-labelled nanocolloid [44]. Indeed, current EANM guidelines for SLNB in breast cancer recommend a mean value for the effective dose of 0.048 mSv [43]. Extensive calculations performed at the Memorial Sloan Kettering Cancer Center have confirmed the safety of SLNB by reporting an effective dose of around 0.2 mSv [51].

It should be noted that adoption of SPECT/CT imaging protocols for SLNB will increase both local radiation dose and effective dose due to inclusion of the CT procedure, the dosimetry being dependent upon the CT acquisition parameters selected. A low-dose CT scan with a field of view limited to avoid radiosensitive tissues can help to keep the effective dose to a minimum. For a low-dose CT scan for attenuation correction, an effective dose of 2.4 mSv per single bed position has been reported [52]. The total exposure in such cases is the emission-generated dose plus the transmission-generated dose [44].

#### Foetal exposure

Pregnant patients may be offered SLNB after careful counselling with regard to the safety and efficacy of the procedure [41]. The maximum value for foetal absorbed dose has been calculated to be 0.013 mSv following the injection of 18.5 MBq [51]. This dose is equivalent to that received by the mother from 1 day of natural background radiation. According to ICRP publications, the risk to the foetus is considered negligible for investigations exposing a foetus to < 1 mSv [53]. Thus, there is no evidence to omit SNLB protocol for radiation safety concerns in pregnant patients, in whom surgery cannot be deferred until after delivery.

#### Lactating women

It has been recommended that breastfeeding should be suspended in nursing mothers for at least 4 h after administration of ^99m^Tc labelled radiopharmaceuticals, since the radiopharmaceutical will be excreted in the breast milk during this period [54]. Any breast milk produced during this time must be discarded.

### Staff dosimetry

#### Staff in operating room

Studies in breast and melanoma patients have determined the mean whole-body dose received by surgical staff to be < 1 μSv per operation [55–57], with a maximum dose to the surgeon of < 2 μSv. The absorbed doses are further minimized when SLNB is performed at 24 h after injection. Monitoring of operating room personnel for occupational exposure during the procedure is therefore unnecessary, and additional shielding is not required. While the pregnant surgeon or scrub nurse requires specific consideration, radiation exposure from participation in fewer than 100 SLNB operations during gestation will remain below the recommended limits for pregnant women [57].

#### Staff in pathology department

Radiation exposure to pathology staff is very low, and should not require badge monitoring. Even personnel performing unusually high numbers of procedures receive radiation doses well below established limits for members of the general population [58].

#### Radiation safety precautions

Labelling specimens as radioactive for transportation to the laboratory is not required, since the surface dose rate is < 5 μGy/h [59].

#### Radioactive clinical waste

Surgical instruments and pathology slides appear to stay at background radiation levels, while measurable contamination of absorptive surgical sponges and other materials used in proximity to the injection site is observed [60]. It is advisable to monitor these materials for contamination, and contaminated materials should be held for an appropriate period of decay-in-storage before disposal [61].

## Future perspectives

The advent of new technological advances (portable gamma cameras, free-hand SPECT devices, dedicated probes and navigation tools) together with preoperative SPECT/CT, has led to the refinement of the original procedures based on conventional gamma camera imaging and handheld gamma probe detection. Moreover, the possibility of combining currently used radiocolloids with other imaging signatures opens new options for further developments [62]. For instance, due to its specific uptake, ^99m^Tc-Tilmanocept can play an important role for SN identification in levels I and II, especially to avoid false-negatives results in floor-of-mouth carcinoma. Another example is illustrated by the use of the hybrid tracer Indocyanine green-^99m^Tc-nanocolloid, which contains a radioactive and a fluorescence signature in a single tracer, making it possible to combine preoperative nuclear medicine imaging with intraoperative radio- and fluorescence guidance [62, 63].

With respect to intraoperative SLN identification, hand-held gamma probes have some limitations. Their intra-operative performance is somewhat operator-dependent and may be impaired by bad count statistics [63]. However, perhaps the biggest limitation is the inability of the gamma probes to provide intraoperative images. Complementary use of blue dyes helps provide visual identification of lymphatic vessels draining from the primary tumour and stain the SLN in about 50% of the cases [62]. Despite the fact that the lymphangiographic blue dyes are not specific for SLNs [28], the combination of preoperative lymphoscintigraphy with the intraoperative use of dye and the gamma probe results in the highest accuracy in SLN identification in melanoma and breast cancer [50]. In these malignancies, blue dyes have been particularly helpful when primary tumour is near the lymphatic basin and the high radiotracer activity deposited locally produces an elevated background, hampering SLN detection by gamma probe counting. However, their use in the head and neck frequently hinders primary tumour visualization of resection margins. The limitations of the use of blue dye, or equivalent fluorescent dyes [28], have been solved with the introduction of ICG-^99m^Tc-nanocolloid, which combines the high sensitivity of gamma detection (due to an increased tissue penetration of radioactivity) with high-resolution intraoperative fluorescence imaging [62, 64–66]. In this scenario, computer technology, three-dimensional rendering systems help to further facilitate intraoperative SLN identification. Combined, these technological advancements are expected to aid the diminishing of false negative rates [63]. It is important to notice that, when intraoperative imaging devices are used, nuclear medicine personnel should be present in the operating room. Table 4 summarises the advantages and limitations of the new perspectives that are under development.

**Table 4:** Future perspectives

Future perspectives				
	Principle	Technique	Advantages	Limitations
Portable gammacameras	Portable device, can be easily moved into small spaces and an articulated arm allows precise imaging. Detector technology with high resolution. Reinforces reliability of detector probe	Properties allow optimal surgical use and increased sensitivity and precision in small spaces	Adaptation of the previous marks to the surgical incision. Confirmation (in real time) of exact localization. Higher resolution (versus complementing lymphoscintigraphy and SPECT/CT) allows detection of SLNs very close to the injection site (frequently missed by preoperative images or gamma probe). Overall reduction in operating time (SLNs found and excised faster)	Availability. Cost
Freehand SPECT	Can be used for pre-surgical and intraoperative navigation and SLN location. It combines acoustic signals or images of a portable camera with a 3D image.	It combines a spatial localization system and two fiducial markers that are attached to the detector probe/camera and the patient. The localization system includes an optical camera and an infrared-based localization device. Enables augmented-reality (AR) and 3D virtual reality displays for surgical navigation.	There is an ability to directly navigate using ether preoperative SPECT/CT images or intraoperative freehand SPECT images. Images can be overlaid onto live video (augmented reality) or be used as navigation targeted (3D virtual reality). The declipse SPECT technology demonstrated potential in oral cavity cancer, melanoma, penile cancer and breast cancer.	Availability. Cost. Specific training is required for data acquisition. Making several readings during surgery can prolong the operation time.
Fluorescence: indocyanine green (ICG)	The angiographic tracer indocyanine green (ICG) has been used in most of the studies	Similar to other vital dyes, ICG rapidly migrates through the lymphatic system with a relatively short detection window. Strict timing is needed. Fluorescence imaging is intuitive and can be used successfully with minimal training.	Precise and fast location of sentinel nodes. More sensitive than blue dye and does not visually stain the surgical field. Wide availability of ICG at low cost (30–80 euro/vial)	Off-label use ICG. Availability and cost fluorescence camera. Visualization and detection of the lymphatic channels and SLNs in obese patients are difficult. It is necessary to plan surgery over a very strict timing schedule in order to timely identify the SLN. Fluorescent signal intensity of ICG is not directly related to the amount of dye used. Penetration of the fluorescence signal through tissues is poor (less than 10 mm). Surgical damage to lymphatic ducts results in contamination of the surgical field.
ICG-99mTc-nanocolloid	By combining a fluorescent and a radioactive signature in a single tracer, traditional radioguidance techniques as well as fluorescence guidance are provided, while preserving the SLN specificity.	Drainage and nodal retention similar to parental radiopharmaceutical allowing combined pre- and intraoperative use. Fluorescence imaging is intuitive, and can be used successfully with minimal training. Hybrid tracers allows surgeons to combine high sensitivity gamma detection with the high resolution of near-infrared imaging.	Preserve preoperative use of lymphoscintigraphy and SPECT/CT for lymphatic mapping. Fluorescence with ICG-99mTc-nanocolloid maintains the SLN specificity and visualizes the same SLN identified during preoperative imaging. Does not visually stain the surgical field. No tracer spillage into the surgical field during resection. Visual surgical guidance becomes available for indications where use of blue dye is undesirable	Availability and cost fluorescence camera. Premixing of ICG and 99mTc-nanocolloid prior to administration.
Opto-nuclear probe	A new hybrid tracing device that combines acoustic gamma and near-infrared fluorescence tracing.	A gamma probe unit has been extended with two optical fibres, one for ICG excitation and one for the detection of the ICG fluorescence emission.	Precise and fast location of sentinel nodes using both radioactive and fluorescent signatures. Ability to utilize the increased spatial resolution of fluorescence detection. Also compatible for combined use of radiotracers and ICG.	No fluorescence images, only tracing. Additional hardware cost to allow for optical tracing.
4D holographic and immersive imaging	Virtual or augmented reality devices applied to the SLNB technique.	Simplified reconstruction and visualisation.	Allows improved planning, monitoring, and training.	Availability. Cost
Gestural control of images	Gestural control devices integrated to the operating theatre imaging systems.	Gestural control devices integrated to the operating theatre imaging systems.	The surgeon can navigate through the images from within the clean area during the surgical intervention. It improves understanding of the images in difficult cases.	Availability. Cost

## Issues requiring clarification

### Diseases or previous treatments that affect lymphatic drainage

Tuberculosis, lymphoma, or previous interventions (radiotherapy and/or surgery) within the neck can distort the normal lymphatic pathways and give rise to unexpected LN uptakes. However, current opinion suggests that lymphoscintigraphy is useful in mapping on aberrant drainage, particularly in cases where the disrupted lymphatic pathways can cause metastasis to occur in unexpected locations [67, 68].

### FDG PET/CT

Due to the insufficient negative predictive value, FDG PET/CT does not replace SLNB in oral cancer. Additionally, due to the lack of specificity, it cannot differentiate malignant from inflammatory lymph nodes, the latter being highly prevalent in the head and neck area.

### Number of injections

The optimal number of injections is four. However, depending on each individual case the number of injections can be decreased or increased.

### Non-migration or non-visualisation of the lymph nodes

This is a rare eventuality. If SPECT/CT is negative, reinjection might be considered [33, 39].

### Depth of invasion

The risk of metastasis increases with tumour depth, but there is no upper or lower limit that can accurately predict the patient’s nodal status [15, 69]. At present, there are no recommendations for neck management based on depth, but this may change with further data.

### Patients with widespread field change

Occasionally, large areas of the oral cavity contain unstable epithelium. These patients are not good candidates for SNB, as the periphery of unstable mucosa is indistinct and it is difficult to assess the potential tumour margin for injection of the tracer.

### Patients who require free flap reconstruction

Neck access is required for vessel anastomosis in patients who require free flap reconstruction. If the SLNB were positive, then a completion neck dissection could put the vascular pedicle at risk. In the future, it might be feasible to schedule the SLNB procedure around 10 days before resection of the primary tumour, so that completion neck dissection can be included in the surgery if required.

### Staged SLNB

Although performing SNB some weeks after resection of the primary tumour is suitable for melanoma, the same results do not appear to be reliable in oral cancer: (a) the cervical lymph nodes become enlarged in the period following surgery and an SLN harvest may retrieve multiple reactive nodes > 2 cm, (b) histopathological processing of large nodes increases laboratory cost, and (c) ‘injection around the scar’ may not accurately reflect the true drainage of the tumour once a surrounding margin has also been excised.

### New tracers

The characteristics of ^99m^Tc-labelled radiotracers are presented in Table 5.

**Table 5:** Characteristics of ^99m^Tc-labelled radiotracers

Agent	Particle size (nm)	
	Maximum	Mean
Sulphur colloid (Sulphur Colloid®)	350–5000 (see text)	100–220 (filtered)
Antimony trisulphide (Lymph Flo®)	80	3–30
Sulphide nanocolloid (Lymphoscint®)	80	10–50
Nanocolloidal albumin (Nanocoll® and NanoTOP®)	100	5–80
Rhenium sulphide nanocolloid (Nanocis®)	500	50–200
ICG-99mTc-Nanocolloid	100	5–80
Tin colloid	800	30–250
Labelled dextran	800	10–400
Hydroxyethyl starch	1000	100–1000
Stannous phytate	1200	200–400
Tilmanocept (Lymphoseek®)	About 7 (equivalence)	About 7 (equivalence)
